# In Vitro Inhibition of *Cryptosporidium parvum* Infection by the Olive Oil Component Oleocanthal

**DOI:** 10.3390/pathogens14101002

**Published:** 2025-10-03

**Authors:** M. Nguele Ampama, Dominik Hanke, Zahady D. Velásquez, Nadine B. Wäber, Carlos Hermosilla, Anja Taubert, Sybille Mazurek

**Affiliations:** 1 Institute of Parasitology, Justus Liebig University Giessen, 35392 Giessen, Germany; zahady.velasquez@vetmed.uni-giessen.de (Z.D.V.); carlos.r.hermosilla@vetmed.uni-giessen.de (C.H.); anja.taubert@vetmed.uni-giessen.de (A.T.); 2 Institute of Veterinary Physiology and Biochemistry, Justus Liebig University Giessen, 35392 Giessen, Germany; dominik.hanke@vetmed.uni-giessen.de (D.H.); nadine-bianca.waeber@vetmed.uni-giessen.de (N.B.W.); sybille.mazurek@vetmed.uni-giessen.de (S.M.)

**Keywords:** *Cryptosporidium parvum*, oleocanthal, mTOR, glycolysis, glutaminolysis, serinolysis, physioxia

## Abstract

Human cryptosporidiosis caused by the zoonotic apicomplexan parasite *Cryptosporidium parvum* represents a neglected and re-emerging poverty-related disease. *C. parvum* possesses minimalistic metabolic capacities and highly depends on its intestinal epithelial host cell for intracellular replication. Based on previous results showing that glycolysis and glutaminolysis inhibition diminished *C. parvum* replication in vitro, we here investigated the impact of the olive oil component oleocanthal on *C. parvum* infection in HCT-8 cells under physioxia (5% O_2_) and hyperoxia (21% O_2_). Oleocanthal targets a broad spectrum of regulatory molecules, amongst which mTOR represents a master regulator of glycolysis and glutaminolysis. Using a host cell pre-treatment as well as a pre- and post-infection treatment protocol, 5 µM oleocanthal reduced *C. parvum* infection rates between 51% and 94%. Host cellular metabolic conversion rates linked oleocanthal-induced inhibition of *C. parvum* infection with an impairment in glutaminolysis, representing an important metabolic pathway in intestinal cells. The principal involvement of mTOR in *C. parvum* inhibition was confirmed by another mTOR-inhibitor (PP242, 0.5 µM), which also reduced *C. parvum* infection by 70–77%. Given that oleocanthal is not a selective mTOR inhibitor, we assume that this compound drives a multi-target-based inhibition of asexual *C. parvum* replication, amongst which mTOR is addressed.

## 1. Introduction

Human cryptosporidiosis is caused by the protozoan parasite *Cryptosporidium parvum* (*C. parvum*) and represents a significant public health concern worldwide. Cryptosporidiosis results in enteritis, often accompanied by catarrhal diarrhoea, abdominal cramps, nausea, vomitus, and hyperthermia. In immunocompromised patients, human cryptosporidiosis can lead to severe chronic and sometimes fatal outcomes. Therefore, human cryptosporidiosis is still considered the second-leading aetiology of diarrhoea-related mortality in children and is responsible for almost a million deaths per year [1]. In addition, *C. parvum* highly affects livestock neonates (mainly calves but also goat kids and lambs), causing significant economic losses mainly in the cattle industry worldwide [1,2]. In the case of calf infections, halofuginone treatments are usually applied in a “metaphylactic” treatment scheme (i.e., all calves of the stock are treated, independent of their actual infection status) but often are ineffective. For immunoprophylaxis in calves, a GP40-based, passive immunization against *C. parvum* (Bovilis^®^ Cryptium^®^, MSD Animal Health, Madison, NJ, USA) was approved in 2023 for use in pregnant heifers or cows in Europe. In humans, so far, the only FDA-approved drug for cryptosporidiosis treatment, i.e., nitazoxanide, cannot be applied in young children and has proved ineffective in immunosuppressed patients [3]. The emergence of drug-resistant *C. parvum* strains underscores the need for alternative novel therapeutics [4]. New effective anti-cryptosporidial drugs are therefore urgently needed for both humans and bovines.

Intracellular replication of *C. parvum* takes place in small intestinal epithelial cells (IEC) of humans and animals. Within its fast division cycles, *C. parvum* undergoes two asexual merogonies followed by a sexual gamogony in IEC, resulting in infective sporulated oocysts carrying four sporozoites, which are shed into the environment. Exogenous sporulated *C. parvum* oocysts are considered highly resistant and are usually ingested orally by humans or other mammalian hosts. After oocyst ingestion, sporozoites are released in the gut and must invade IEC as host cells, thereby forming a parasitophorous vacuole (PV), positioning the parasite in its intracellular but extracytoplasmic location [5].

In general, cell proliferation poses high demands on the metabolism of a cell, which must provide both necessary energy and cell building blocks, such as nucleic acids, proteins and lipids in sufficient quantities for daughter cell formation. Due to its reduced own genome, the metabolic repertoire of *C. parvum* is very limited [6]. On the plus side, the genome of *C. parvum* includes all enzymes of glycolysis as well as glucose transporters, which facilitate the uptake of glucose from its host cell. In addition, *C. parvum* is able to split glucose phosphate from amylopectin under the catalysis of glycogen phosphorylase [7]. Together, this enzymatic equipment allows *C. parvum* to use glucose phosphates to generate energy via the glycolytic pathway. In fact, in newborn calves, *C. parvum* was shown to compete with IEC for glucose and to impair systemic glucose supply [8]. Based on the importance of glycolysis in *C. parvum* metabolism, in the last decade, key enzymes of glycolysis, such as hexokinase (HK) and lactate dehydrogenase (LDH), were identified as promising therapeutic targets for the treatment of cryptosporidiosis [9,10,11,12]. On the minus side, *C. parvum* is only equipped with a reduced mitochondria-like organelle called “mitosome”. As a result, *C. parvum* lacks the citric acid cycle, the electron transport chain and a functional ATP synthase [13,14,15]. In addition, regarding synthetic pathways, *C. parvum* lacks de novo biosynthetic pathways for amino acids, nucleosides, and fatty acids [13]. As consequence of these metabolic deficiencies, the obligate intracellular proliferation of *C. parvum* highly depends on its host cell, providing the necessary nutrients as well as metabolic intermediates and products for energy regeneration and synthesis of essential cellular building blocks. Measuring metabolic conversion rates in cell culture supernatants of *C. parvum*-infected human ileocecal colorectal adenocarcinoma cells (HCT-8) and bovine small intestinal explants (BSIE), we previously observed that *C. parvum* infection not only increased glucose consumption and lactate production but also glutaminolytic conversion rates of their host cells [12,16]. Consistently, in HCT-8 cells, not only the inhibition of HK, LDH and mitochondrial pyruvate carrier (MPC) but also of glutaminase, the first enzyme within glutaminolysis, led to an inhibition of *C. parvum* infection. Similarly, pharmacological inhibition of the monocarboxylate transporters 1, 2 and 4 (MCT 1, 2, 4), which export lactate, an end-product of both glycolysis and glutaminolysis as well as serinolysis, showed significant anti-cryptosporidial effects [12].

One of the critical key regulators of glycolysis and glutaminolysis in general, and of the metabolic processes targeted by the inhibition of HK, LDH, MCTs, MPC and glutaminase is the mammalian target of rapamycin (mTOR) (Figure S1) [17,18,19,20,21,22]. Vice versa mTORC1, the complex of mTOR with Raptor, is activated by glutaminolysis and a high energy status via inhibition of adenosine monophosphate kinase (AMPK) in cells [23] (Figure S1). Accordingly, glucose and energy deprivation and the associated activation of AMPK, acidification of the cytosol as well as inhibition of glycolysis and glutaminolysis —all metabolic interventions induced by inhibition of HK, LDH, MPCs, MCTs, and glutaminase—have been shown to have a negative impact on mTORC1 [17,18,19,22,23,24] (Figure S1). Therefore, we wondered whether inhibition of mTOR as one of the master regulators of glycolysis and glutaminolysis could also be an effective approach for inhibiting *C. parvum* replication. Inhibitors of mTOR have been intensively investigated for therapeutic use in cancer, but also arthritis, type 2 diabetes, neurodegenerative diseases and others [25,26,27,28]. Meanwhile, several generations of mTOR inhibitors have been developed to optimize therapeutic applications in humans. Amongst these, oleocanthal, a natural component of olive oil, and PP242 (a synthetic second-generation mTOR inhibitor, also known as Torkinib) were described to bear inhibitory mTOR capacity. Thus, PP242 was reported as a potent ATP-competitive mTORC1 and mTORC2 inhibitor [29] whilst both, oleocanthal and PP242 have been shown to efficiently reduce mTOR phosphorylation [30,31].

Within an organ, the metabolism of cells is influenced not only by the nutrient supply but also by oxygen conditions of the cellular environment. The influence of oxygen pressure also applies to parasite-infected host cells within the small intestine [12,16]. In the small intestine, the apical villi tips (closest to the intestine lumen) experience oxygen concentrations between 5–11%, while in the crypts, oxygen concentrations decrease to 1% [32]. Referring to the high impact of oxygen concentrations on cellular metabolism and to mimic intestinal conditions as physiologically as possible, we conducted current in vitro experiments in the presence of 5% oxygen (=physioxia for the intestinal epithelium) in addition to 21% oxygen, commonly used in classical laboratory conditions, but signifying hyperoxia when compared to the physiological oxygen conditions in the small intestine. When considering the situation in cattle industry, where all calves of a flock are treated as soon as *C. parvum*-induced diarrhea occurs, i.e., irrespective of the actual individual infection status, we chose a first protocol in which host cells were exclusively pre-treated by inhibitors for 24 h and *C. parvum* infections were then performed in absence of inhibitors (host cell pre-treatment, mimicking prophylactic treatments in calve stock scenario) (Figure S2). Since oleocanthal showed superiority over PP242 in inhibiting *C. parvum* infection in this protocol and is also a naturally occurring substance in olive oil, we focused on oleocanthal in all subsequent experiments and additionally introduced a pre- and post-infection treatment protocol, mirroring a metaphylactic/post-infection treatment in the calf stock scenario. In the pre- and post-infection treatment protocol, oleocanthal was added to the cell culture medium 24 h prior to *C. parvum* infection, washed out before infection to avoid direct effects on parasite stages, and re-administered again for 45 h after three hours of *C. parvum* sporozoite supplementation with consecutive wash-out of non-invaded parasites, as described in Figure S2 [12].

Here, we show for the first time that the olive oil component oleocanthal efficiently inhibits *C. parvum* infection in vitro under physioxic and hyperoxic O_2_ conditions, both when administered before and after *C. parvum* infection and as pure host cell pre-treatment. Metabolic conversion rates measured in HCT-8 cell culture supernatants indicate impairment of glutaminolysis by oleocanthal in *C. parvum*-infected HCT-8 cells. Glutaminolysis, an important metabolic pathway in intestinal cells, is positively regulated by mTORC1 via Myc and glutaminase. In turn, glutamine and high glutaminolysis conversion rates have an activating effect on mTORC1 (Figure S1) [17,20,23,24,28].

## 2. Materials and Methods

### 2.1. Host Cell Culture

Permanent human HCT-8 cells (cells isolated from a large intestine adenocarcinoma; ATCC-CCL-244, LGC Standards GmbH, Wesel, Germany) were maintained at 37 °C and 5% CO_2_ using sterile RPMI 1640 cell culture medium (R0883, Sigma-Aldrich, Darmstadt, Germany) supplemented with 2 mM glutamine (Sigma-Aldrich, Darmstadt, Germany), 10% (*v*/*v*) fetal bovine serum (FBS; S0115, Biochrom AG, Berlin, Germany), 100 UI penicillin and 0.1 mg streptomycin/mL (both Sigma-Aldrich, Darmstadt, Germany). Based on our previous observation that *C. parvum* infection rates were drastically reduced in HCT-8 cells with more than 7 passages after thawing, we only used HCT-8 cells between passage 2 and 7 in current experiments. Infection rates, immunofluorescence and metabolic signature assays were performed in 24-well plates containing 13 mm diameter glass coverslips (Thermo Fisher Scientific, Waltham, MA, USA) coated with fibronectin (1: 400, Sigma-Aldrich, F1141-2MG, Darmstadt, Germany). Given that we worked with the permanent cell line HCT-8, only one biological replicate was used. Thus, all replicates indicated in the single experiments signify technical replicates maintained independently in separate wells.

### 2.2. Inhibitors and Treatment Protocols

Olecanthal was purchased from Sigma-Aldrich (SMB00810-5MG, St. Louis, MO, USA) and resolved in DMSO to a final concentration of 16.43 mM. PP242 was purchased from Cyman Chemical (13643, Ann Arbor, MI, USA) and dissolved in DMSO to a final concentration of 4 mM.

For the “host cell pre-treatment protocol” the following experimental setting was established (see Figure S2): HCT-8 cells were simultaneously seeded in the presence of 21% O_2_ or 5% O_2_ and cultured for approximately 3 days until a sub-confluent cell monolayer. Thereafter, the medium was changed, and the cells were treated with inhibitors for 24 h (2.5 µM and 5.0 µM oleocanthal, 0.25 µM and 0.5 µM PP242). Control cells were mock-treated with 0.1% (*v*/*v*) DMSO, which corresponded to the DMSO concentration supplemented in the medium of the highest oleocanthal (5 µM) or PP242 (0.5 µM) concentrations. After washing-off the inhibitors (three washings with prewarmed 1× PBS), *C. parvum* oocysts (see 2.4.) were added into fresh medium without inhibitors to infect HCT-8 cells [multiplicity of infection (MOI) = 1:2, oocysts–cells]. After 3 h, non-invaded extracellular sporozoites were washed off, and cells were cultivated for a further 45 h in fresh medium in the absence of the inhibitors. At the end of this cultivation period (=48 h p. i.), cells were fixed, and infection rates and intracellular development were estimated.

In case of the “pre- and post-infection treatment protocol”, the same steps as described for the “host cell pre-treatment” protocol were performed, including the 3-h infection period. Thereafter, non-invaded extracellular sporozoites were washed out, inhibitors were re-administered, and cells were cultivated for a further 45 h in fresh medium in the presence of 5 µM oleocanthal. At the end of this cultivation period (=48 h p. i.), the cells were subjected to measurements of the infection rates and metabolic fluxes (Figure S2). In both the “host cell pre-treatment” and the “pre- and post-infection treatment” protocol, untreated *C. parvum*-infected HCT-8 cells and inhibitor-treated uninfected HCT-8 cells were co-cultured in parallel, serving as infection and treatment controls, respectively. All control cells without inhibitor treatment were mock-treated with 0.1% (*v*/*v*) DMSO, which corresponds to the DMSO concentration in the cell culture media of cells treated with 5 µM oleocanthal or 0.5 µM PP242.

### 2.3. XTT Tests

For XTT tests (Cat. No. 39904, SERVA Electrophoresis GmbH, Heidelberg, Germany), HCT-8 cells were seeded in RPMI 1640 cell culture medium (R0883, Sigma-Aldrich, Darmstadt, Germany) supplemented with 2 mM glutamine (Sigma-Aldrich, Darmstadt, Germany), 10% (*v*/*v*) FBS (S0115, Biochrom AG, Berlin, Germany), 100 UI penicillin and 0.1 mg streptomycin/mL (both Sigma-Aldrich, Darmstadt, Germany) at a density of 1 × 10^4^ cells per well in a 96-well plate (Greiner AG, Kremsmünster, Austria). Each experimental condition was performed in three replicates. After confluency, cells were pre-treated with each drug (oleocanthal: 10 µM; 5 µM; 2.5 µM and 1.25 µM; PP242: 1.0 µM; 0.5 µM; 0.25 µM and 0.125 µM), diluted in cell culture medium (0.5% (*v*/*v*) DMSO) for 72 h at 5% CO_2_ and 37 °C in accordance with the total inhibitor incubation times in the pre- and post-infection treatment protocol. XTT reagent was reconstituted by adding 100 µL of Activation Reagent (PMS) to 5 mL of XTT reagent. 50 µL XTT working solution was added to each well, including blanks, and the samples were incubated at 37 °C and 5% CO_2_ for 2 h. Thereafter, signals were quantified in a Varioskan TM Flash Multimode Reader (Thermo Fisher Scientific, Waltham, MA, USA) at 450–500 nm.

### 2.4. C. parvum Oocyst Excystation and Host Cell Infection

Sporulated oocysts of *C. parvum* were purchased at Waterborne Inc. (New Orleans, LA, USA, Iowa isolate, P102C). Excystation was performed according to Tandel et al. [33] and initiated by treatment of sporulated oocysts with 8.25% (*v*/*v*) sodium hypochlorite for 10 min on ice. Thereafter, oocysts were washed thrice in sterile PBS by centrifugation (18,000× *g*, 10 min). Oocysts were resuspended in excystation medium [0.8% (*v*/*v*) sodium taurocholate, 2.5% (*v*/*v*) trypsin in sterile PBS] and incubated for 10 min at 37 °C. Thereafter, oocysts were washed thrice in sterile PBS (18,000× *g*, 10 min). Finally, the pellet was resuspended in sterile RPMI 1640 cell culture medium (R0883, Sigma-Aldrich, Darmstadt, Germany) supplemented with 2 mM glutamine (Sigma-Aldrich, Darmstadt, Germany), 1% (*v*/*v*) FBS (S0115, Biochrom AG, Berlin, Germany), 100 UI penicillin and 0.1 mg streptomycin/mL (both Sigma-Aldrich, Darmstadt, Germany). Pre-excysted oocysts were counted and used to infect HCT-8 cells at a multiplicity of infection (MOI) of 1:2 (oocysts–cells) [33]. All data result from two independent infection experiments.

### 2.5. Immunofluorescence Microscopic Detection of Intracellular C. parvum Stages

HCT-8 cells were propagated as described above and in Figure S2 and fixed with paraformaldehyde (4% (*v*/*v*); Merck KGaA, Darmstadt, Germany) for 15 min. After three washings in sterile 1× PBS, the samples were incubated in a blocking/permeabilization solution [sterile 1× PBS with 3% (*w*/*v*) BSA and 0.3% (*v*/*v*) Triton X-100; all Sigma-Aldrich, Darmstadt, Germany] for 1 h at room temperature (RT). Thereafter, samples were stained with fluorescein-conjugated *Vicia villosa* (1:1000; FL-1231-2, Vector Laboratories, Newark, CA, USA) and phalloidin (1:3000; ab176757, Abcam Limited, Cambridge, UK) at RT in a humidified chamber (1 h in complete darkness) as described elsewhere [12,16]. The samples were then washed again thrice in sterile 1× PBS and mounted with an anti-fading mounting medium solution with DAPI (Fluoromount G-DAPI, 495952, Thermo Fisher Scientific, Waltham, MA, USA).

### 2.6. Fluorescence Image Acquisition

Fluorescence confocal images were acquired with a ReScan Confocal instrumentation (RCM 1.1 Visible, Confocal, Amsterdam, The Netherlands) with a fixed 50 μm pinhole and combined with a Nikon Eclipse Ti2-A inverted microscope equipped with a motorized z-stage unit (DI1500, Nikon, Tokyo, Japan). The RCM unit was connected to a Toptica CLE laser with the following excitation modes: 405/488/561/640 nm. Images were taken by an sCMOS camera (PCO edge) using a CFI Plan Apochromat X60 lambda-immersion oil objective (NA 1.4 / 0.13; Nikon, Tokyo, Japan). The instrument was operated by the NIS-Elements software (version 5.11). Images were acquired via a z-stack optical series with a step size of 0.1 microns to cover all structures of interest.

Infection rate quantification was performed in epifluorescence-acquired images (BZ-X800 microscope, Keyence, Osaka, Japan). Sixteen fields of view were blinded and randomly taken to count the total number of cells based on DAPI staining (cell nuclei) and the number of infected cells (positive for VVL). Images were first segmented using the Otsu thresholding algorithm, preserving identical brightness and contrast conditions for each data set within one experiment. The total number of cells was obtained using the Fiji plugin “Analyzed particles” with a size threshold of 10 μm [34].

### 2.7. Quantification of Metabolic Conversion Rates in Host Cell Culture Supernatants

HCT-8 cells were grown in 24-well plates (Greiner Bio-One GmbH, Frickenhausen, Germany) pre-coated with fibronectin (1:400, F1141-2MG, Sigma-Aldrich, Darmstadt, Germany) at 37 °C and 5% CO_2_ atmosphere. Cells were cultured, drug-treated and infected with viable *C. parvum* sporozoites as described in the pre- and post-infection treatment protocol in Figure S2. At the end of the 45 h cultivation period, cell supernatants were collected, centrifuged (400× *g*, 10 min, 4 °C), immediately frozen in liquid nitrogen and stored at −80 °C until measurements. In parallel, the corresponding numbers of cells/well for each supernatant sample were counted for normalization purposes. For metabolite concentration assessment, frozen samples were heated (15 min, 95 °C) and centrifuged (8000× *g*, 10 min). Concentrations of glucose, pyruvate, lactate, glutamine, glutamate, serine, alanine and aspartate were determined using a Respons 920 bench-top analyzer (DiaSys Diagnostic Systems GmbH, Holzheim, Germany), as previously described [12,16,35]. The conversion rates of individual metabolites were determined in [nmol/(h × 10^4^ cells)], referring to medium samples without cells, which were cultured in parallel to the wells with cells during the 45 h lasting cultivation period, starting with the medium change after parasite infection.

### 2.8. Statistical Analysis

The data were expressed as the mean ± SD of six independent experiments (n = 6). For infection rate experiments, the normality of the data sets was evaluated by a Shapiro–Wilk normality test. When two groups were compared, an unpaired *t*-test was performed. When more than three data sets were compared, a one-way ANOVA test followed by a Tukey multiple comparisons post-test was applied. For metabolic signature data, a Kruskal–Wallis test was performed, followed by Dunn’s multiple comparisons post hoc tests. The effect of the oxygen concentration was tested by using a two-way ANOVA test followed by a Šidák multiple comparisons post-test (Table S2). All statistical analyses were performed using GraphPad Prism 9.3.1 software (GraphPad Software Inc., San Diego, CA, USA), applying a significance level of 5% (α = 0.05).

## 3. Results

### 3.1. Effects of Oleocanthal and PP242 Treatments on C. parvum Infection

**Figure 1:**
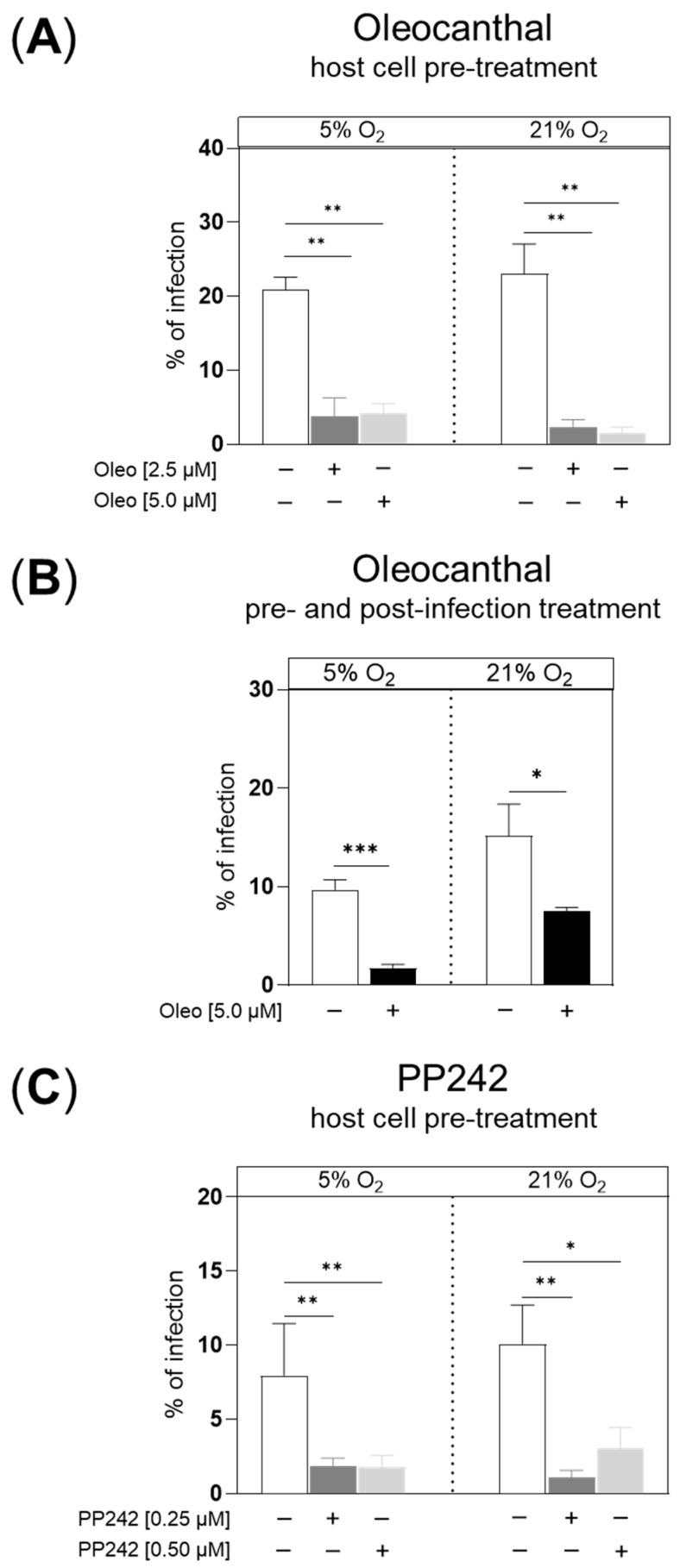
Effects of oleocanthal and PP242 treatments on *C. parvum* infection under physioxia (5% O_2_) and hyperoxia (21% O_2_) conditions. Confluent HCT-8 cells were cultivated for 24 h in the presence of the respective inhibitor, infected for 3 h and cultivated for another 45 h p. i. without inhibitor = host cell pre-treatment (**A**,**C**) or cultivated for another 45 h in the presence of the inhibitor = pre- and post-infection treatment (**B**) (Figure S2). Both inhibitors were tested in parallel at 5% and 21% O_2_ conditions. As read-out of inhibitor effects, infection rates were estimated microscopically at 48 h p. i. after fixing and staining the cells by *Vicia villosa* to detect *C. parvum* stages and by DAPI to detect host cell nuclei. The total number of infected host cells was counted and divided by the total number of host cells in the same field of view and graphed as a percentage of infected cells (infection rate). Graph bars represent the mean of six replicates ± SD. *: *p* < 0.05%, **: *p* < 0.01%, ***: *p* < 0.001%.

In preceding dose-finding experiments, 2.5 µM and 5 µM for oleocanthal and 0.25 µM and 0.5 µM for PP242 were identified as suitable test concentrations, which did not affect the vitality of HCT-8 cells under both oxygen conditions tested (i.e., 5% O_2_ and 21% O_2_) (Figure S3). By restricting experimentation to these concentrations, we attempted to ensure that measured effects were exclusively due to *C. parvum* infection and did not reflect inhibitor-driven effects on host cells. Overall, oleocanthal treatments effectively reduced *C. parvum* infection rates at 48 h p. i., irrespective of dosage and treatment scheme (Figure 1A,B). Referring to the impact of O_2_ conditions, no O_2_-driven effects on infection rates were detected when pretreating the cells with oleocanthal or PP242 (Figure 1A,C). However, in the pre- and post-infection treatments scheme (Figure 1B), 5 µM oleocanthals proved less effective under 21% O_2_ (*p* = 0.0005, Table S1 and Figure 1B).

To verify oleocanthal-driven effects, we applied another inhibitor, i.e., PP242, which was also described to efficiently reduce phosphorylation of mTOR [29,30] by pre-treating HCT-8 cells with two doses (0.25 µM and 0.5 µM) 24 h prior to *C. parvum* infection. PP242 also significantly reduced *C. parvum* infection rates in HCT-8 cells (Figure 1C) by 77% at 48 h p. i. in the presence of 5% O_2_, regardless of the inhibitor concentration used. When the highest doses of oleocanthal and PP242, which showed no effect on host cell vitality in the previous dose-finding study, were compared under hyperoxia, the reduction in the *C. parvum* infection rate with PP242 was slightly lower (70%) than with oleocanthal, which inhibited the *C. parvum* infection rate by 94%. (Figure 1A,C). Given that the current work focused on oleocanthal, we additionally tested the efficacy of this compound by applying the pre- and post-infection treatment experimental protocol (i.e., treatment 24 h before the infection and 45 h after the infection), which may better mimic the field situation in calf stocks of dairy cattle with unknown individual infection status. Applying this treatment scheme, 5 µM oleocanthal also significantly reduced *C. parvum* infection rates by 82% at physioxia and by 51% at hyperoxia conditions at 48 h p. i. (Figure 1B).

**Figure 2:**
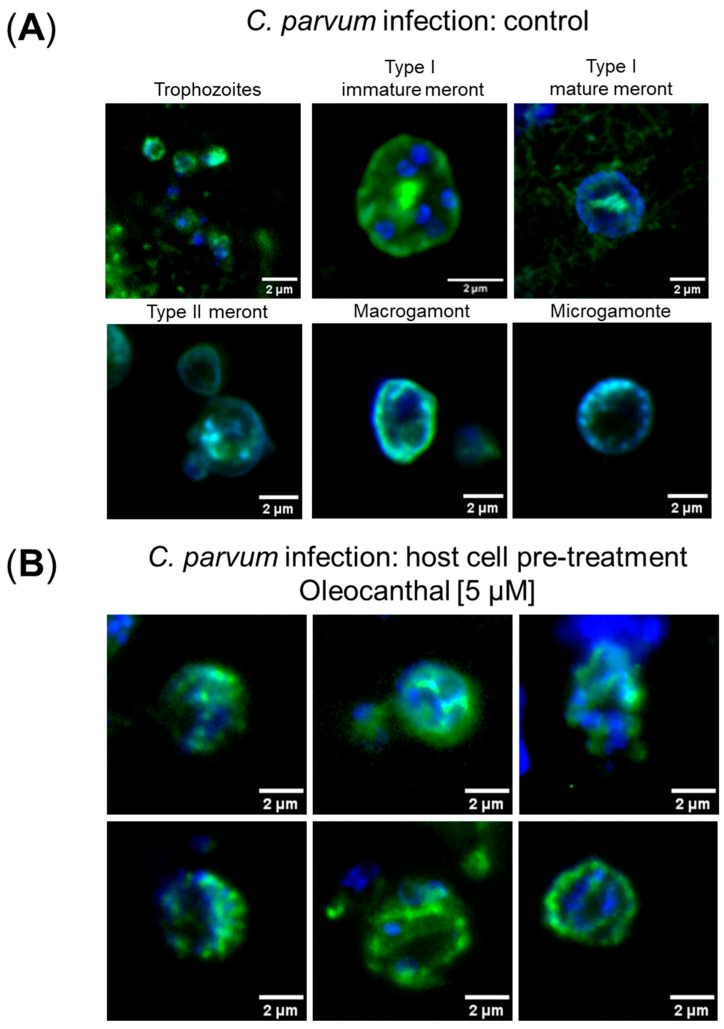
Impact of oleocanthal on the intracellular development of *C. parvum.* Cells were cultivated and treated according to the host cell pre-treatment protocol (Figure S2). The intracellular development of *C. parvum* was monitored microscopically after *Vicia villosa* (VVL, green) staining; cell nuclei were labelled by DAPI (blue). (**A**) Mock-treated *C. parvum*-infected HCT-8 cells (=controls). (**B**) *C. parvum*-infected HCT-8 cells after host cell pre-treatment with 5 µM oleocanthal. Scale bar: 2 µm.

To control whether oleocanthal-driven findings were due to either parasitostatic or parasiticidal effects, we microscopically monitored *C. parvum* development in oleocanthal pre-treated *C. parvum*-infected HCT-8 cells and inhibitor-free *C. parvum*-infected HCT-8 cells. As expected, in *C. parvum*-infected controls lacking oleocanthal treatment, we found all classical intracellular developmental stages and were able to illustrate trophozoites, type I and II meronts, macrogamonts and microgamonts by confocal microscopy. Likewise, in oleocanthal pre-treated HCT-8 cells, *C. parvum* development seemed not abrogated at the early trophozoite stage, but a high proportion of developing meronts II were severely affected in their morphology, showing signs of degradation, thereby potentially indicating oleocanthal-derived parasiticidal effects of oleocanthal at this stage (Figure 2).

### 3.2. Impact of Oleocanthal Treatment on the Metabolic Signature of C. parvum-Infected HCT-8 Cells in Physioxia and Hyperoxia

**Figure 3:**
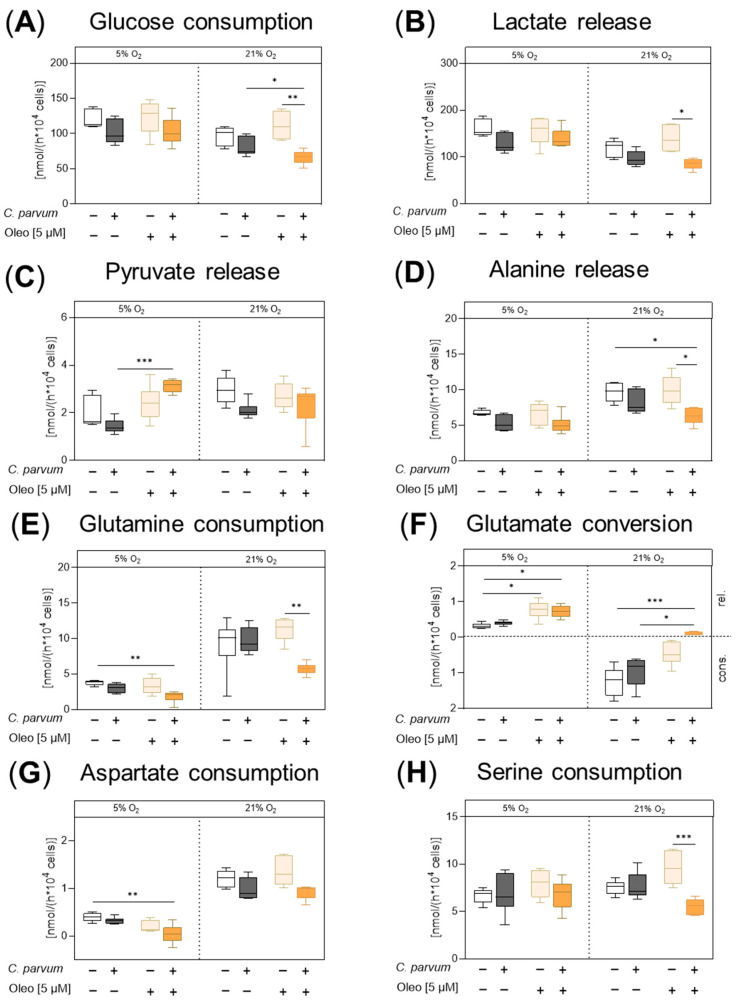
Effects of oleocanthal treatments on the metabolic signatures of uninfected and *C. parvum*-infected HCT-8 cells under physioxia (5% O_2_) and hyperoxia (21% O_2_) conditions. Cells were treated according to the pre- and post-infection treatment protocol (Figure S2). Metabolic conversion rates were measured in the cell culture supernatants in order to analyse glucose consumption (**A**), lactate release (**B**), pyruvate release (**C**), alanine release (**D**), glutamine consumption (**E**), glutamate conversion (**F**), aspartate consumption (**G**), and serine consumption (**H**). Bar colours represent each treatment: n.i. (white), *C. parvum*-infected cells (dark grey), oleocanthal-treated cells (light orange), and *C. parvum*-infected cells after oleocanthal treatment (dark orange). Cons. = consumption. Rel. = release. Bars represent the mean of six replicates ± SD. *: *p* < 0.05%, **: *p* < 0.01%, ***: *p* < 0.001%.

**Figure 4:**
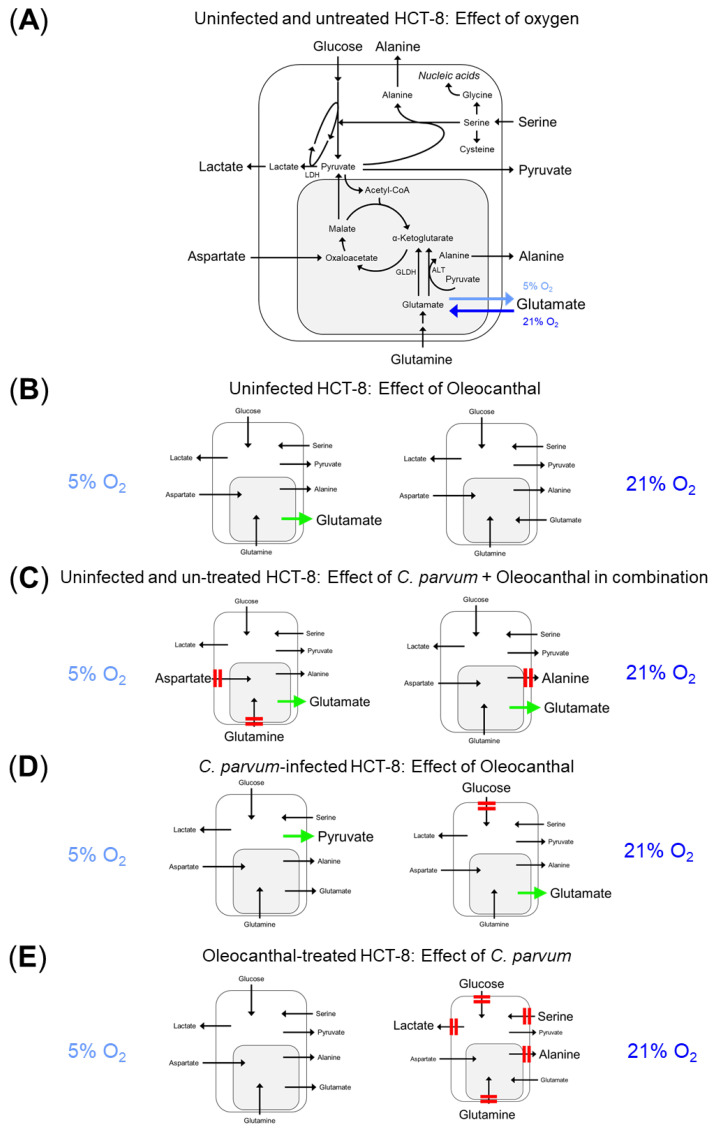
Metabolic scheme to outline the impact of oleocanthal treatment on glycolytic, glutaminolytic and serinolytic conversion rates in uninfected and *C. parvum*-infected HCT-8 cells under physioxia (5% O_2_) and hyperoxia (21% O_2_) conditions. Comparison with Figure 3: (**A**) Detailed metabolic scheme of the abstracted schemes (**B**–**E**). Black arrows correspond to control cells without infection and oleocanthal treatment (=white bars in Figure 3). Blue arrows represent differences between 5% and 21% O_2_ in uninfected and untreated control cells. Light blue arrow: 5% O_2_; dark blue arrow: 21% O_2_. LDH = lactate dehydrogenase, GLDH = glutamate dehydrogenase, ALT = alanine aminotransaminase. (**B**) Comparison of light orange bars (uninfected, oleocanthal-treated HCT-8 cells) versus white bars (uninfected and untreated controls) in Figure 3. (**C**) Comparison of dark orange bars (*C. parvum*-infected and oleocanthal-treated HCT-8 cells) versus white bars (uninfected and untreated controls) in Figure 3. (**D**) Comparison of dark orange bars (*C. parvum*-infected and oleocanthal-treated HCT-8 cells) versus dark gray bars *(C. parvum*-infected HCT-8 without oleocanthal treatment) in Figure 3. (**E**) Comparison of dark orange bars (*C. parvum*-infected and oleocanthal-treated HCT-8 cells) versus light orange bars (uninfected, oleocanthal-treated HCT-8 cells) in Figure 3. Black arrows indicate unchanged conversion rates between the respective cell groups. Red double lines indicate a reduction in the respective conversion rate by the treatment compared. Bold green arrows indicate an increase in the respective conversion rate by the treatment compared.

The impact of oleocanthal on *C. parvum*-infected HCT-8 cells and uninfected controls was characterized by measuring the conversion rates of selected nutrients and metabolic products of glycolysis, glutaminolysis and serinolysis in cell culture supernatants of HCT-8 cells treated with oleocanthal or DMSO vehicle according to the pre- and post-infection treatment protocol (Figure S2). Treatment with 5 µM oleocanthal induced a significant increase in the release of glutamate into the medium under physioxic conditions (5% O_2_) in uninfected control cells, which indicates that less glutamate was fueled into the citric acid cycle and glutaminolysis was thus impaired (Figure 3F: 5% O_2,_ light orange bars versus white bars, Figure 4B). In contrast to physioxia, under hyperoxic cultivation conditions (21% O_2_), untreated control cells consumed glutamate from the medium (Figure 3F: 5% O_2_: white bars versus 21% O_2_:white bars, Figure 4A) and oleocanthal treatment reduced glutamate consumption of uninfected HCT-8 cells by tendency (Figure 3F: 21% O_2_, light orange bars versus white bars). When *C. parvum*-infected and oleocanthal-treated HCT-8 cells were compared with completely untreated control cells (without both *C. parvum* infection and oleocanthal treatment) the conversion rates point to an impairment of glutaminolysis under both oxygen conditions, as reflected by a significant decrease in glutamine and aspartate consumption and increase in glutamate release under physioxic conditions (Figure 3E–G: 5% O_2_, dark orange bars versus white bars, Figure 4C), and a significant reduction in the release of alanine and a shift from glutamate consumption to release under hyperoxia (Figure 3D, F 21% O_2_, dark orange bars versus white bars, Figure 4C). When the effect of the combination of oleocanthal treatment and *C. parvum* infection was statistically compared to the individual treatments (*C. parvum* infection or oleocanthal treatment), under physioxia, pyruvate release significantly increased when *C. parvum*-infected HCT-8 cells were treated with oleocanthal while the release of lactate and alanine remained unchanged (Figure 3B–D: 5% O_2_, dark orange bars versus dark gray bars, Figure 4D), which together indicated that part of the pyruvate synthesized intracellularly, e.g., via glycolysis, glutaminolysis and serinolysis, was released as such without conversion to lactate or alanine or infiltration in the citric acid cycle (Figure 4A). Under hyperoxia, in *C. parvum*-infected host cells, oleocanthal treatment was accompanied by a significant decrease in glucose consumption and a shift from glutamate consumption in the absence of oleocanthal to release in presence of oleocanthal, which together point at an impairment of glycolysis and glutaminolysis (Figure 3A,F: 21% O_2_, dark orange bars versus dark gray bars, Figure 3D). Likewise, under hyperoxia, when *C. parvum*-infected oleocanthal-treated HCT-8 cells were compared with oleocanthal-treated uninfected HCT-8 cells, a significant decrease in glucose consumption, lactate release, glutamine consumption, serine consumption, as well as alanine release was observed (Figure 3A,B,D–H: 21% O_2_, dark orange bars versus light orange bars, Figure 4E), indicating an impairment of glycolysis, glutaminolysis and serinolysis. As expected, a change in O_2_ conditions affected the metabolic signatures of *C. parvum*-infected HCT-8 cells. Hence, glucose consumption (*p* < 0.0001), lactate production (*p* < 0.0001) and glutamate conversion (*p* < 0.0001) was found enhanced at 5% O_2_, whilst alanine release (*p* < 0.0001), glutamine consumption (*p* < 0.0001), and aspartate conversion (*p* < 0.0001) was lowered at these conditions when compared to 21% O_2_ (Table S2).

## 4. Discussion

Based on our previous findings demonstrating that inhibitors of glycolysis and glutaminolysis, as well as of MCTs, known to be involved in the export of lactate out of host cells, inhibited *C. parvum* infection in vitro [12], we wondered whether an inhibition of mTOR, as one of the master regulators of glycolysis and glutaminolysis, might also be a suitable therapeutic target for the treatment of cryptosporidiosis. Besides being a key regulator of glycolysis, glutaminolysis, autophagy, immune response, survival, proliferation and growth, mTOR itself is impaired by acidification of the cytosol, nutrient and energy deficiency and inhibition of glutaminolysis [17,18,19,20,22,23,24,28], which are all metabolic processes that were also influenced by inhibition of HK, LDH, MCTs and glutaminolysis in our previous study [12] and in the study of Eltahan et al. [10].

When searching for mTOR inhibitors in the literature to test our hypothesis we came across oleocanthal, which was shown to reduce mTOR phosphorylation by more than 50% when supplemented in a concentration of 10 µM into the medium of MDA-MB-231 cells for 72 h [31]. Oleocanthal is a natural compound of olive oil, which has received significant interest in the search for naturally derived compounds with pharmacological qualities. Thus, diverse beneficial effects of oleocanthal on the level of antioxidant, anticancer, anti-inflammatory, antibacterial, neuroprotective, and antiplatelet aggregation activities were demonstrated (see reviews of El Haouari et al. 2020, Jannati et al., 2025 [36,37] and references therein).

*C. parvum* represents the most important zoonotic *Cryptosporidium* species with high global prevalence in the main host, the cattle (calves), causing high losses in the cattle industry worldwide [38,39]. Human infections mainly affect young children in underdeveloped countries and immunocompromised patients [40]. In both host systems, effective treatments are currently lacking. In the bovine host, the indication of halofuginone treatments—even though largely ineffective—is to reduce diarrhea in neonatal calves of dairy farms with a history of cryptosporidiosis. In calf stocks, usually all animals are treated at a time, irrespective of their actual infection status. Given that not all calves are infected at the same time point, treatments may therefore be given before and after *C. parvum* infection. To consider this classical field situation, which may also apply for young children in underdeveloped countries, we here used two different treatment protocols by either treating host cells for 24 h before *C. parvum* infection or by applying treatments at both the pre-infected and infected status.

Overall, low-dose (2.5 µM) oleocanthal pre-treatments of host cells showed a high anti-cryptosporidial efficacy by reducing *C. parvum* infection rates by 82% and 90%, when being cultivated at 5% and 21% O_2_, respectively. Doubling the oleocanthal dosage to 5 µM did not significantly improve antiparasitic efficacy. Confocal microscopic analyses of *C. parvum* developmental stages illustrated phenotypic effects at the meront level, thereby matching with anti-cryptosporidial activity, but also indicating that parasite development was not in all cases abrogated before meront formation. Noteworthy, one of the limitations of the HCT-8 cell model is its inability to fully support the life cycle of *C. parvum,* since the fertilization step is blocked in this permanent cell line [33]. Consequently, the current data identify oleocanthal as an interesting compound acting on asexual multiplication, whilst effects on sexual replication cannot be deduced from this specific host cell model.

To investigate the general suitability of mTOR as a target for inhibiting *C. parvum* infection, we also investigated the impact of PP242, a second compound for which mTOR inhibition is described in the literature as a mode of action [30], on *C. parvum* infection, applying the same experimental procedure (supplementation of inhibitors only before infection) as in the case of oleocanthal. Indeed, PP242 treatments proved effective against *C. parvum* infection (ranging from 70–90% inhibition; 5% and 21% O_2_) when inhibitor concentrations were used that did not affect the vitality of host cells (0.25 µM and 0.5 µM), thereby verifying a potential role of mTORC1 inhibition in impairing *C. parvum* infections. Accordingly, mTOR knockdown by siRNAs limited *C. parvum* burden in HCT-8 cells in vitro [41].

Given that physiological O_2_ in vivo concentrations in the intestinal setting (1–11% O_2_) highly vary from those typically applied in research laboratories (21% O_2_), we performed current experimentation at both 5% and 21% O_2_ conditions. In general, when applying oleocanthal treatments for 24 h before infection as well as for 45 h after infection (pre- and post-infection treatment), a significant reduction of *C. parvum* infection rates was also achieved, thereby confirming a principal anti-cryptosporidial effects of oleocanthal. However, the current data indicated no effects of the oxygen concentration on the efficacy of oleocanthal (or PP242) pretreatments even though the metabolic signatures significantly changed with oxygen conditions. Hence, exclusively in the pre- and post-infection treatment scheme, oleocanthal proved significantly less effective at 21% O_2_ when compared to 5% O_2_. So far, we do not have an explanation for this finding.

Besides being useful for the detection of antiparasitic effects, the pre- and post-infection treatment protocol also allowed the estimation of inhibitor-driven impact on the host cell metabolism in the presence of the parasite. Overall, the assessment of metabolic signatures of *C. parvum*-infected cells in the presence and absence of oleocanthal revealed that oleocanthal-driven effects on glycolytic, glutaminolytic and serinolytic conversion rates were indeed influenced by oxygen concentration in an infection status-dependent manner. In uninfected control cells experiencing 5% O_2_ and 5 µM oleocanthal, the increase in glutamate production, combined with unchanged glutamine consumption rates, points to an oleocanthal-driven impairment of the glutaminolytic conversion rates. In line, in astrocytes, amyloid-β-induced downregulation of the glutamate transporter could be reversed by 7-day treatment of the cells with 5 µM oleocanthal [42]. Besides glutaminolysis, all other conversion rates measured under physioxic conditions in uninfected HCT-8 cells (glucose, serine and aspartate consumption, lactate, pyruvate and alanine production) were not significantly affected when oleocanthal was supplemented in a concentration of 5 µM, which did not affect the vitality of the host cells. Apart from Bataresh et al., 2017 [42], no further data on oleocanthal-driven effects on in vitro conversion rates of glycolysis, glutaminolysis or serine and alanine metabolism were found in the literature at the time point of writing this manuscript. From the above-mentioned findings in uninfected physioxic HCT-8 cells, it can be concluded that under physioxic conditions, oleocanthal host cell pre-treatment for 24 h prior to infection resulted in host cells with diminished glutaminolysis activity, a status that was already proven as adverse for *C. parvum* replication, since glutaminase inhibitors effectively reduced infection rates [12]. Conversely, *C. parvum* infection was also inhibited under hyperoxic conditions in the host cell pre-treatment approach, even though metabolic conversion rates in uninfected hyperoxic HCT-8 cells did not change during oleocanthal treatment in comparison to untreated control cells. From this finding, it can be concluded that in hyperoxia—and presumably also in physioxia—there are further—in this study not yet identified—effects of oleocanthal on host cells, which affect *C. parvum* replication.

The observed impairment of glutaminolysis found under both oxygen conditions, when the conversion rates in *C. parvum*-infected and oleocanthal-treated HCT-8 cells were compared with the conversion rates in uninfected control cells without oleocanthal treatment indicated an influence of *C. parvum* on the oleocanthal effect and vice versa.

Basically, the observed reduction in glutaminolysis under both oxygen conditions in oleocanthal-treated, *C. parvum*-infected HCT-8 cells is consistent with a possible involvement of mTOR in the oleocanthal-induced impairment of *C. parvum* infection in HCT-8 cells (Figure S1), whereby, in principle, both regulatory directions are thinkable: an impairment of glutaminolysis by oleocanthal-induced inhibition of mTOR but also an inactivation of mTOR by oleocanthal-induced inhibition of glutaminolysis [17,19,22]. Interestingly, inhibition of glutaminolysis by the glutaminase inhibitor CB-839 sensitized ovarian cancer cells to PP242 treatment [43], the same mTOR inhibitor which also blocked *C. parvum* infection in this study. In line to a possible involvement of mTOR in the inhibition of *C. parvum* infection, an oleocanthal-driven reduction in mTOR phosphorylation by more than 50% was demonstrated in MDA-MB-231 breast cancer cells treated for 72 h with 10 µM oleocanthal [31].

Moreover, recently Yang et al., 2023 [44] described an inhibition of intracellular *C. parvum* proliferation by the mTOR inhibitor rapamycin in HCT-8 cells, thereby correlating this effect with autophagy. Phosphorylated and activated mTOR inhibits autophagy, a lysosomal degradation process involved in the elimination of pathogens from cells [45]. However, pathogens, including parasites, may escape autophagy and/or can use host cell autophagy to provide themselves with nutrients generated by the degradation of host cell molecules, promoting their proliferation. Accordingly, two independent studies [46] in Caco-2 cells and [44] in HCT-8 cells revealed on the one hand that *C. parvum* may induce autophagy by inhibiting mTOR phosphorylation and enhancing the autophagic flux. Likewise, mTOR knockdown by siRNAs promoted autophagy, increased the autophagic flux and apoptosis and significantly lowered the parasite burden in HCT-8 cells [41]. On the other hand, *C. parvum* may escape autophagy-derived elimination by promoting successful intracellular survival via EGFR/PI3K/AKT pathway activation [44].

Since the first reports on anti-inflammatory and anti-oxidant effects of oleocanthal via inhibition of cyclooxygenase 1 and 2 and 5-LOX in 2005 [47,48] a multitude of medical applications and modes of action of oleocanthal have been discovered. The most prominent applications are inflammations, neurodegenerative diseases and cancer. Selected signaling pathways from the very extensive orchestra of published oleocanthal targets are: (i) the HGF- c-MET axis (=hepatocyte growth factor cellular mesenchymal–epithelial transcription factor-axis) which leads to an impairment of the RAF-MEK-ERK signaling pathway, the PI3K-AKT-mTOR signaling pathway and NF-kappa B, as well as STAT3 and (ii) Interleukin 6-dependent Janus kinase 1/2 (JAK 1/2 which does also impair STAT3 phosphorylation and activation or (iii) peroxisome proliferator-activated receptor gamma (PPARγ) (Figure S1) (reviewed in [37,49,50]). The oleocanthal-induced suppression of STAT3 phosphorylation shows interesting overlaps with the above-discussed relationships between the observed oleocanthal effects on glutaminolysis and the mTOR signaling pathway, thereby suggesting STAT3 as an alternative or additional target of the oleocanthal-induced inhibition of *C. parvum* infection (Figure S1). Similar to mTOR, phosphorylation and activation of STAT3 lead to increased glutamine conversion via induction of Myc [51] and like mTOR, the STAT3 signal cascade is in turn activated by high glutamine conversion [52]. Treatment of colorectal cancer cell lines with 15 and 10 µM oleocanthal led to a dose-dependent inhibition of JAK 1/2 and STAT3 activation, respectively [53]. Of note, a role for the STAT3 signaling pathway in the development of ileocecal adenocarcinomas has recently been described in the context of murine *C. parvum* infections [54].

In summary, the suppression of *C. parvum* by PP242 in this study and by rapamycin in the study by Yang et al., 2023 [44]. indicates a potential impact of mTOR inhibition on *C. parvum* proliferation. However, due to the broad spectrum of action in the case of oleocanthal, it cannot be concluded that inhibition of a single target, such as mTOR or STAT3 alone induced the suppression of *C. parvum* infection in our cell model. Rather, the diverse applications and the broad spectrum of proven oleocanthal targets suggest multiple points of attack in different signaling pathways and target functions of the respective treated cells.

## 5. Conclusions and Outlook

Here, we identified oleocanthal—a natural component of olive oil—as a promising compound for inhibiting *C. parvum* asexual intracellular replication in vitro using two different treatment protocols, namely (*i*) treatment before and after infections, as well as (*ii*) treatment exclusively before infection, which might be relevant concerning the in-field situation in the livestock industry. *C. parvum* inhibition by oleocanthal treatments was demonstrated in both protocols, not only under classical laboratory conditions of 21% O_2_ but also under physiological oxygen concentrations present in the small intestine of humans and bovines (~5% O_2_).

In the next step, future studies in suitable animal models (i.e., calves, lambs, goat kids) are needed to verify the applicability and efficacy of oleocanthal prophylactic and medical treatments of *C. parvum* infections in vivo.

The improved understanding of the broad spectrum of action of oleocanthal led to several clinical studies on the potential benefits of dietary oleocanthal supplementation in the form of olive oil in the prevention of neurodegenerative diseases, rheumatoid arthritis and cancer. Some of these studies emphasize benefits for patients [55,56,57]. However, olive oils can have varying oleocanthal contents ranging from 0.2 mg/kg to 1200 mg/kg (reviewed in [49,58]). Even when using olive oils with high oleocanthal content, the low absorption upon oral administration demonstrated in rats, as well as hydration, hydrogenation, hydroxylation and oxidation of oleocanthal in phase 1 metabolism, and glucuronidation in phase 2 metabolism [59,60] in addition to the bitter and irritant taste of olive oil with high polyphenol content, these represent obstacles for the oral application of oleocanthal in pure olive oil formulations for the treatment of *C. parvum* infections in animals and humans. As an alternative to olive oil formulation, Tajmim et al. [61] presented two new oral oleocanthal formulations, which significantly decreased Aβ plaque deposition and STAT3 phosphorylation in 5xFAD transgenic mice at an oral dosage of 10 mg oleocanthal / kg in microcrystalline cellulose capsule shells (6 times a week over a period of 6 month). Of note, oleocanthal concentrations used in the current study ranged between 0.75 mg/L (=2.5 µM) and 1.5 mg/L (=5 µM), which correspond to approximately 1/6 and 1/13, respectively, of the oleocanthal concentration orally administered to 5× DAD mice [61]. To date, additional strategies on further pharmaceutical formulations, such as phytosome formulations, are studied to improve the bioavailability of oleocanthal in oral applications (reviewed in [37]).

## Data Availability

The original contributions presented in this study are included in the article. Further inquiries can be directed at the first author.

## Supplementary Materials

The following supporting information can be downloaded at: https://www.mdpi.com/article/10.3390/pathogens14101002/s1, Figure S1: Interactions between selected signal cascades targeted by oleocanthal and enzymes of glycolysis and glutaminolysis, whose inhibition has been associated with downregulation of *C. parvum* infection. Figure S2: Schemes describing the experimental protocols of host cell pre-treatment and pre- and post-infection treatment of HCT-8 cells in this study. Figure S3: Analysis of oleocanthal- and PP242-driven effects on HCT-8 cells viability. Table S1: Kruskal–Wallis effect size η^2^ calculation for metabolic signature data, corresponding to Figure 3. Table S2: Statistical analysis of the oxygen effect on experiments shown in Figure 1A–C, and Figure 3A–H.

## Abbreviations

The following abbreviations are used in this manuscript:

BSA	Bovine serum albumin
DAPI	4′,6-diamidino-2-phenylindole
DMSO	Dimethyl Sulfoxide
HK	Hexokinase
LDH	Lactate dehydrogenase
MCT	Monocarboxylate transporters
MOI	Multiplicity of infection
mTOR	Mammalian target of rapamycin
Oleo	Oleocanthal
PBS	Phosphate-Buffered Saline
PP242	2-(4-Amino-1-isopropyl-1H-pyrazolo [3,4-d]pyrimidin-3-yl)-1H-indol-5-ol, Dihydrate
SD	Standard deviation
STAT3	Signal transducer and activator of transcription 3
