# Supplementary material for: In Vitro Inhibition of Cryptosporidium parvum Infection by the Olive Oil Component Oleocanthal
Source: Pathogens. 2025 Oct 3;14(10):1002. doi: 10.3390/pathogens14101002 (PMC12566851; doi:10.3390/pathogens14101002)
Supplement: Supplementary file 1 [file pathogens-14-01002-s001.zip › Manuscript_Pathogens_Supps_final.pdf]

Supplementary Material

Inhibition of *Cryptosporidium parvum* infection by the olive oil component Oleocanthal

M. Nguele Ampama[1,2*], Dominik Hanke[2], Zahady D. Velasquez[1], Nadine B. Wäber[2], Carlos Hermosilla[1], Anja Taubert[1] and Sybille Mazurek[2]

[1]Institute of Parasitology, Justus Liebig University Giessen, 35392 Giessen, Germany

[2]Institute of Veterinary Physiology and Biochemistry, Justus Liebig University Giessen, 35392 Giessen, Germany

* Correspondence: Mireille Nguele Ampama honorine.nguele-ampama@vetmed.uni-giessen.de

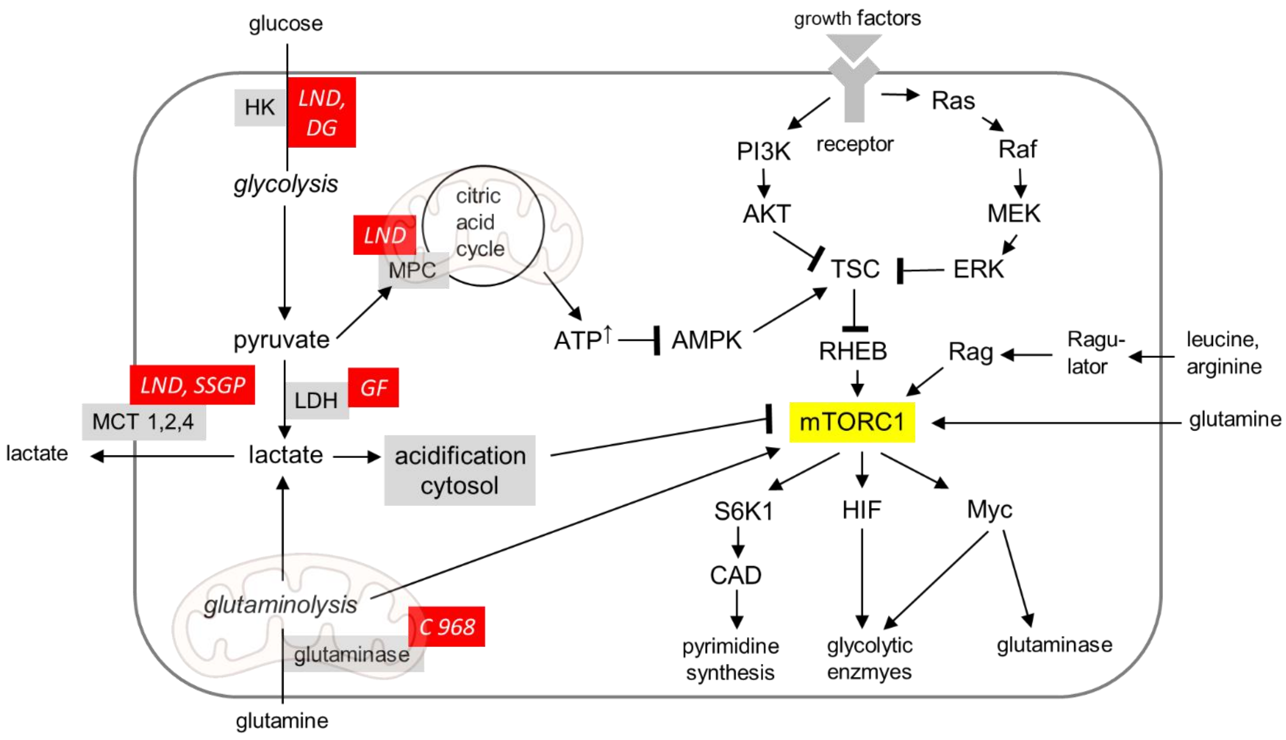

Figure S1: Scheme of selected metabolic pathways regulated by mTORC1 and regulating mTORC1. In the TORC1 complex, mTOR activates pyrimidine synthesis, glycolysis, and glutaminolysis, thereby supporting cell metabolism and cell proliferation. Conversely, mTORC1 is inhibited by glucose deprivation, inhibition of glutaminolysis, energy deprivation and the associated activation of AMPK, as well as cytosol acidification [1-8]. Induction of each of these metabolic deficiency situations by inhibiting hexokinase (HK) by lonidamine (LND) and 2-deoxy-D-glucose (DG), lactate dehydrogenase (LDH) by galloflavin (GF), monocarboxylate transporter (MCT) by LND and syrosingopine (SSGP), mitochondrial pyruvate carrier (MPC) by LND and glutaminase by compound 968 (C968) resulted in a significant reduction of the *C. parvu*m infection rate in previous in vitro studies (marked in red within the scheme) [9,10], which raises the question of whether *C. parvum* infection can also be impaired by inhibiting the master regulator mTOR in the host cells. AKT = protein kinase B; CAD = carbamoyl-phosphate synthetase 2; ERK = extracellular signal-regulated kinase; HIF-1 = hypoxia inducible factor 1; MEK = mitogen-activated protein kinase kinase; mTOR = mammalian target of rapamycin; mTORC1 = complex of mTOR with raptor; PI3K = phosphatidyl inositol 3-P-kinase; Raf = rapidly accelerated fibrosarcoma = proto-oncoprotein); Rag = rat sarcoma virus; RAS = rat sarcoma = proto-oncoprotein; S6K1 = ribosomal protein S6 kinase 1.

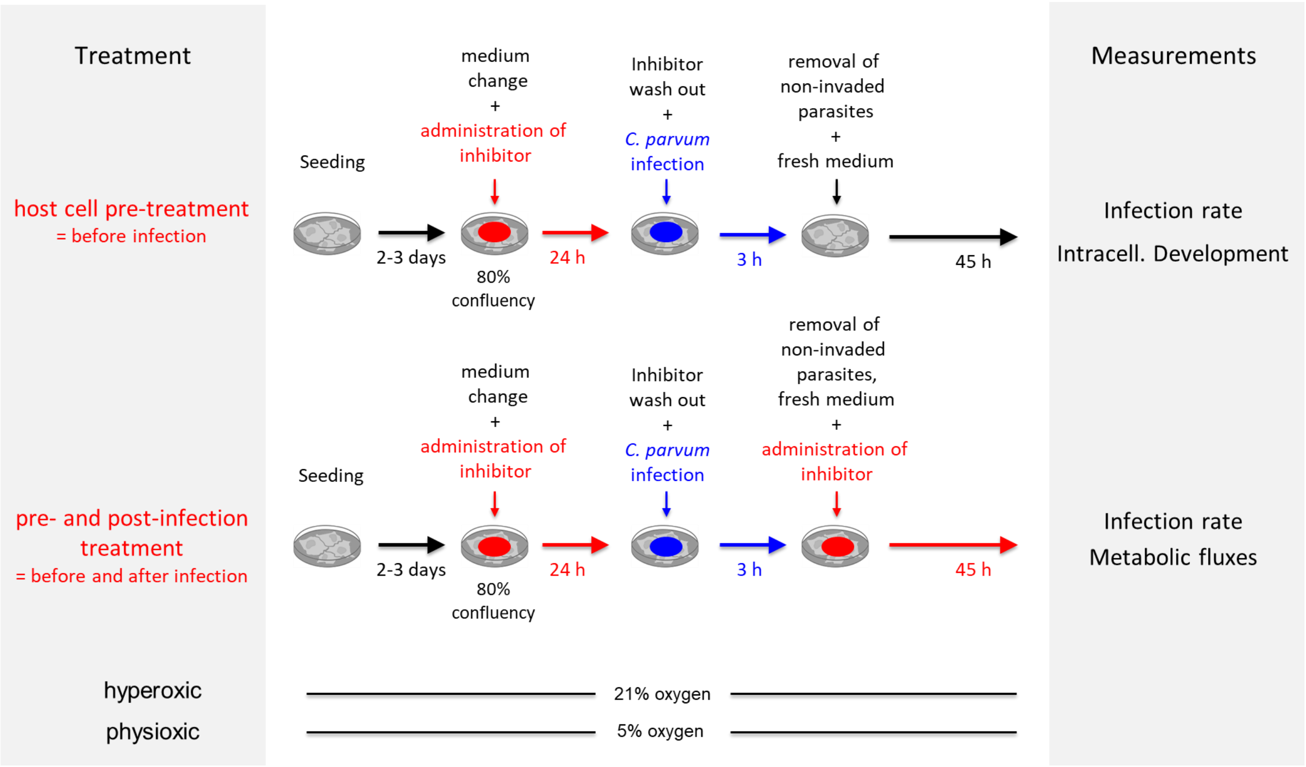

Figure S2: Schemes describing the experimental protocols of host cell pre-treatment and pre- and post-infection treatment of HCT-8 cells in this study. HCT-8 cells were divided into two groups: cultivated in presence of 5% O_2 (= physioxia) or cultivated in presence of 21% O_2 = (hyperoxia) and cultivated for 3 days in presence of 5% or 21% O_2, respectively to reach subconfluence. The assigned O_2 condition was maintained throughout the subsequent protocol. Thereafter, the medium was changed, and the cells were treated with the inhibitors or the vehicle (= DSMO) for 24 h. After medium change excysted *C. parvum* sporozoites were added in the absence of the inhibitors to infect HCT-8 cells (MOI 1:2). Three hours p. i. non-invaded extracellular sporozoites were washed-out and cells were cultivated for further 45 h in fresh medium in the absence of inhibitor (= host cell pre-treatment protocol) or in the presence of the inhibitor (= pre- and post-infection treatment protocol).

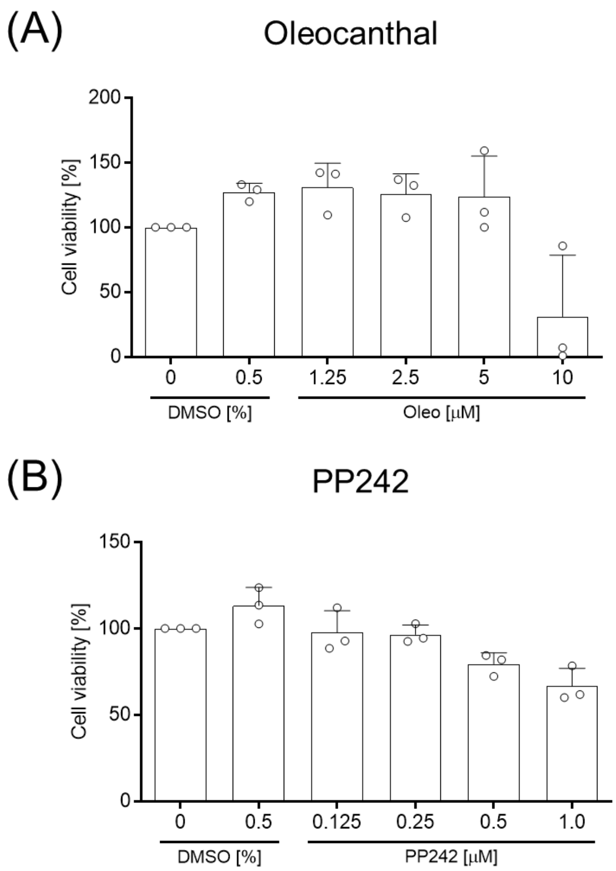

Figure S3: Analysis of oleocanthal- and PP242-driven effects on HCT-8 cells viability. HCT-8 cells were treated with oleocanthal (Oleo; 1.25 – 10 µM) or PP242 (0.125 – 1.0 µM). Mock-treated controls, which were treated with the DMSO concentration supplemented at the highest concentration of oleocanthal and PP242 (= 0.5% (*v*/*v*) DMSO), and un-treated HCT-8 cells, which were cultivated in the absence of inhibitors and DMSO (= 0% DMSO) were propagated in parallel for controls. After 72 hours of treatment, cells were subjected to a commercial XTT test (SERVA Electrophoresis GmbH, Heidelberg, Germany) and signals were quantified in a Varioskan TM Flash Multimode Reader at 450-500 nm.

Supplementary References

1. Durán, R.V.; Oppliger, W.; Robitaille, A.M.; Heiserich, L.; Skendaj, R.; Gottlieb, E.; Hall, M.N. Glutaminolysis activates Rag-mTORC1 signaling. *Molecular cell* **2012**, *47*, 349-358, doi:10.1016/j.molcel.2012.05.043.
2. Fonseca, B.D.; Diering, G.H.; Bidinosti, M.A.; Dalal, K.; Alain, T.; Balgi, A.D.; Forestieri, R.; Nodwell, M.; Rajadurai, C.V.; Gunaratnam, C.; et al. Structure-Activity Analysis of Niclosamide Reveals Potential Role for Cytoplasmic pH in Control of Mammalian Target of Rapamycin Complex 1 (mTORC1) Signaling. *J Biol Chem* **2012**, *287*, 17530-17545, doi:10.1074/jbc.M112.359638.
3. Li, Y.; Chen, C.; Yao, F.; Su, Q.; Liu, D.; Xue, R.; Dai, G.; Fang, R.; Zeng, J.; Chen, Y.; et al. AMPK inhibits cardiac hypertrophy by promoting autophagy via mTORC1. *Arch Biochem Biophys* **2014**, *558*, 79-86, doi:10.1016/j.abb.2014.06.023.
4. Paquette, M.; El-Houjeiri, L.; Pause, A. mTOR Pathways in Cancer and Autophagy. *Cancers (Basel)* **2018**, *10*, doi:10.3390/cancers10010018.
5. Fan, H.; Wu, Y.; Yu, S.; Li, X.; Wang, A.; Wang, S.; Chen, W.; Lu, Y. Critical role of mTOR in regulating aerobic glycolysis in carcinogenesis (Review). *Int J Oncol* **2021**, *58*, 9-19, doi:10.3892/ijo.2020.5152.
6. Bodineau, C.; Tome, M.; Courtois, S.; Costa, A.S.H.; Sciacovelli, M.; Rousseau, B.; Richard, E.; Vacher, P.; Parejo-Perez, C.; Bessede, E.; et al. Two parallel pathways connect glutamine metabolism and mTORC1 activity to regulate glutamoptosis. *Nature communications* **2021**, *12*, 4814, doi:10.1038/s41467-021-25079-4.
7. Szwed, A.; Kim, E.; Jacinto, E. REGULATION AND METABOLIC FUNCTIONS OF mTORC1 AND mTORC2. *Physiol Rev* **2021**, *101*, 1371-1426, doi:10.1152/physrev.00026.2020.
8. Kim, J.; Guan, K.L. mTOR as a central hub of nutrient signalling and cell growth. *Nat Cell Biol* **2019**, *21*, 63-71, doi:10.1038/s41556-018-0205-1.
9. Eltahan, R.; Guo, F.; Zhang, H.; Zhu, G. The Action of the Hexokinase Inhibitor 2-deoxy-d-glucose on *Cryptosporidium parvum* and the Discovery of Activities against the Parasite Hexokinase from Marketed Drugs. *J Eukaryot Microbiol* **2019**, *66*, 460-468, doi:10.1111/jeu.12690.
10. Vélez, J.; Velasquez, Z.; Silva, L.M.R.; Gärtner, U.; Failing, K.; Daugschies, A.; Mazurek, S.; Hermosilla, C.; Taubert, A. Metabolic Signatures of *Cryptosporidium parvum*-Infected HCT-8 Cells and Impact of Selected Metabolic Inhibitors on *C. parvum* Infection under Physioxia and Hyperoxia. *Biology (Basel)* **2021**, *10*, doi:10.3390/biology10010060.
